# Bacterial community in saline farmland soil on the Tibetan plateau: responding to salinization while resisting extreme environments

**DOI:** 10.1186/s12866-021-02190-6

**Published:** 2021-04-20

**Authors:** Yi Qiang Li, Ying Hui Chai, Xu Sheng Wang, Li Ying Huang, Xi Ming Luo, Cheng Qiu, Qing Hai Liu, Xiang Yu Guan

**Affiliations:** 1 School of Ocean Sciences, China University of Geosciences (Beijing), Beijing, 100083 China; 2 Laboratory division, Eighth Medical Center of Chinese People’s Liberation Army General Hospital, Beijing, 100000 People’s Republic of China; 3 Institute of Agricultural Quality Standards and Testing, Tibet Academy of Agriculture and Animal Husbandry Sciences, Lhasa, 850000 China; 4 Beijing Key Laboratory of Water Resources and Environmental Engineering, China University of Geosciences (Beijing), Beijing, 100083 China

**Keywords:** Saline, Tibetan plateau, Metagenomics, Microbial community, Resistance mechanism

## Abstract

**Background:**

Salinization damages the health of soil systems and reduces crop yields. Responses of microbial communities to salinized soils and their functional maintenance under high salt stress are valuable scientific problems. Meanwhile, the microbial community of the salinized soil in the plateau environment is less understood. Here, we applied metagenomics technology to reveal the structure and function of microorganisms in salinized soil of the Tibetan Plateau.

**Results:**

The diversity of composition and function of microbial community in saline soil have changed significantly. The abundances of chemoautotrophic and acidophilic bacteria comprising *Rhodanobacter*, *Acidobacterium*, *Candidatus* Nitrosotalea, and *Candidatus* Koribacter were significantly higher in saline soil. The potential degradation of organic carbon in the saline soil, as well as the production of NO and N_2_O via denitrification, and the production of sulfate by sulfur oxidation were significantly higher than the non-saline soil. Both types of soils were rich in genes encoding resistance to environmental stresses (i.e., cold, ultraviolet light, and hypoxia in Tibetan Plateau). The resistance of the soil microbial communities to the saline environment is based on the absorption of K^+^ as the main mechanism, with cross-protection proteins and absorption buffer molecules as auxiliary mechanisms in our study area. Network analysis showed that functional group comprising chemoautotrophic and acidophilic bacteria had significant positive correlations with electrical conductivity and total sulfur, and significant negative correlations with the total organic carbon, pH, and available nitrogen. The soil moisture, pH, and electrical conductivity are likely to affect the bacterial carbon, nitrogen, and sulfur cycles.

**Conclusions:**

These results indicate that the specific environment of the Tibetan Plateau and salinization jointly shape the structure and function of the soil bacterial community, and that the bacterial communities respond to complex and harsh living conditions. In addition, environmental feedback probably exacerbates greenhouse gas emissions and accelerates the reduction in the soil pH. This study will provide insights into the microbial responses to soil salinization and the potential ecological risks in the special plateau environment.

**Supplementary Information:**

The online version contains supplementary material available at 10.1186/s12866-021-02190-6.

## Background

The Tibetan Plateau is located at a high altitude (average > 4500 m), with severe cold and low oxygen levels, and it is strongly affected by ultraviolet radiation [1, 2]. Due to global warming, population increases, and the fragility of the Tibetan Plateau environment, various ecological and environmental problems have occurred, such as vegetation degradation, biodiversity decline, desertification, and salinization [3, 4]. Soil salinization is considered one of the most pressing environmental challenges for the world [5, 6]. The continued salinization of scarce agricultural soil resources will have feedback effects on global climate change, as well as detrimentally affecting the already poor living conditions for people on the Tibetan Plateau. However, saline soil and its impact on extreme climate change in this complex and fragile environment have received little attention.

Microorganisms are essential components of the soil ecosystem on the Tibetan Plateau and they play key roles in the health of the ecosystem [7, 8]. Microorganisms adapt to high salinity environment mainly through two mechanisms comprising the synthesis or absorption of organic osmotic agents, and absorbing K^+^ and other inorganic ions to resist osmotic stress [9, 10], thereby maintaining the normal life activities of cells under high osmotic pressure conditions. Meanwhile microbial community also adapt to salinity by adjusting its composition and enhancing interactions [11, 12]. Soil samples from different high salinity regions vary greatly in microbial community structures, and bacteria are more sensitive than fungi [13, 14]. Studies of saline soils throughout the world have shown that salinization affects not only the bacterial community composition but also metabolic functions. Salinity leads to significant decreases in the soil microbial diversity and biomass, reductions in the soil enzyme activities [15, 16], inhibition of bacterial growth and respiration [17], retardation of the organic matter degradation rate and suppression of nitrification [18]. The mechanisms of bacteria resisting high salinity environments consume large amounts of energy, and the organic matter in the soil will be consumed rapidly [19]. Bacteria with autotrophic capacities are likely to have survival advantages in salinity soil, thereby leading to changes in the metabolic functional network for the bacterial community. However, no bacteria are specifically adapted to high-salinity soil environments and it is not easy to find bacterial indicator in salinity soil [20]. The soil microbial community on the Tibetan Plateau has responded to extreme environmental pressures via a unique metabolic mechanism [21, 22]. However, the microbial communities in saline soils at high altitude have not been investigated.

In the sixth century BC, humans mainly settled in the northeast area of the Tibetan Plateau, and they did not extend their agricultural activities to the land higher than 3600 m above sea level in the central and southern Tibetan Plateau until 3500 cal yr B. P [23]. The melting of glaciers and repeated freezing–thawing of permanently frozen soils caused by global warming have partially exposed the glacier-covered mineralized rock layers on the surface of the Tibetan Plateau. In addition, the increased water yield has accelerated the leaching of various minerals in the rock and acid rock drainage into the rivers [24, 25]. Thus, irrigation using river water has resulted in large amounts of sulfate and metal ions being applied to land, leading to salinization of the soil in the study area. The environmental challenges encountered by soil bacterial communities in farmland in the study area include high soil salinity, temperature differences between the day and night, extremely strong ultraviolet radiation, limited oxygen, and other extreme conditions. Thus, bacterial survival under these conditions evolved specific survival strategies. In this study, we will focus on the: (1) characteristics of bacterial community in saline soil on the Tibetan Plateau, and their biogeochemical cycling processes, (2) the mechanisms associated with the responses to multiple environmental pressures, and (3) the potential impacts of bacterial communities in salinized soil on environmental climate.

## Methods

### Soil sampling

The study area is located in Naidong County, Shannan City, Tibet, with an average altitude of 3560 m. Yala Snow Mountain is a natural snow mountain glacier with the highest altitude in the area of 6647 m and it is the main water source. The study area has a temperate monsoon plateau climate and the air is thin. The average annual temperature in Naidong County is 8.8 °C, the average annual pressure is 660.4 hPa, the average annual solar radiation is 6018.9 MJ, and the average annual precipitation is 383.2 mm.

In May 2019, saline soil samples (SA) were collected from farmland near the Zhiqu River that had been planted with barley (Fig. S1), and nonsaline soil samples (CK) were collected as a control from farmland near the Yalong River (Xiangqu) that had also been planted with barley. Five subsamples were collected at each sampling site according to the four corners of a square and the center point, where the side length was about 10 m. The surface 5–20 cm soil layer was collected and each sample was packed in a 50-ml sterile centrifuge tube. The sample tubes were refrigerated with ice packs and returned to the laboratory within 24 h. Each of the subsamples was passed through a 2-mm sieve in the laboratory to remove any stones and plant debris. The five soil subsamples from the same location were mixed to obtain one sample. The mixed saline soil samples were designated as S1, S2, S3, S4, S5, and S6, and the nonsaline soil samples as N1, N2, N3, N4, N5, and N6. Each soil sample was divided into two parts, and one was stored at 4 °C for subsequent chemical tests and experiments in the laboratory. The other was kept at − 20 °C for DNA extraction.

### Geochemical analysis

The soil samples were dried at 55 °C and crushed, before passing through a 2-mm sieve. The soil samples were mixed at a soil: water ratio of 1:5 (w/v), shaken well, and allowed to stand for 48 h. The supernatant was passed through a filter membrane with a pore size of 0.45 μm to prepare the test solution for the experiments. The soil electrical conductivity (EC) value was measured in a suspension with a soil:water ratio of 1:5 (w/v) using a CLEAN Conductivity Tester (CON30; FC Corporation, California, USA). The soil pH value was measured in a suspension with a soil:water ratio of 1:5 (w/v) using a pH meter (PB-10; Sartorius, Goettingen, Germany). The pore-water dissolved nitrate (NO_3_^−^) and sulfate (SO_4_^2−^) contents were analyzed by ion chromatography (DX-120, DIONEX, Bannockburn, IL, USA) [26]. The Total organic carbon (TOC) content (Calculated as carbon dioxide) was confirmed by using a high-frequency infrared Carbon-Sulfur Analyzer (LECO CS744, LECO Corporation, USA). Total nitrogen (TN) was analyzed using a Eurovector elemental analyzer (Isoprime-EuroEA 3000, Milan, Italy). The available nitrogen (AN) contents were determined with the alkaline digestion diffusion method. The total sulfur (TS) contents were measured using the infrared absorption method after high frequency combustion (High-speed Analyzer HWF-900A, Wuxi, China). Other trace metal (loid) s were analyzed by ICP-MS (ThermoFisher X-series, Franklin, MA, USA) and ICP-AES (TJA IRIS-Advantage, Franklin, MA, USA).

### DNA extraction, library construction, and metagenomic sequencing

The total DNA was extracted from each soil sample (0.5 g) using a PowerSoil DNA Extraction Kit (MoBio Laboratories, Carlsbad, CA, USA). The quantity and quality of isolated DNA were evaluated using a NanoDrop spectrophotometer (ND-2000, Thermo Fisher Scientific, Waltham, MA, USA) and agarose gel electrophoresis (Bio-Rad, Hercules, CA, USA), respectively. The extracted DNA was stored at − 20 °C until further analysis, or at − 80 °C for long-term storage.

DNA was fragmented to an average size of about 300 bp using Covaris M220 (Gene Company Limited, China) for paired-end library construction. Paired-end library was prepared by using TruSeq™ DNA Sample Prep Kit (Illumina, San Diego, CA, USA). Adapters containing the full complement of sequencing primer hybridization sites were ligated to the Blunt-end fragments. Paired-end sequencing was performed on Illumina HiSeq3000 platform (Illumina Inc., San Diego, CA, USA) at Majorbio Bio-Pharm Technology Co., Ltd. (Shanghai, China) using HiSeq 3000 PE Cluster Kit and HiSeq 3000 SBS Kits according to the manufacturer’s instructions (www.illumina.com). The 3′ and 5′ ends were stripped (https://github.com/jstjohn/SeqPrep) and low-quality reads were removed (https://github.com/najoshi/sickle). The software SOAPdenovo (http://soap.genomics.org.cn, Version 1.06) was employed to assemble short reads and K-mers were tested for each sample. The software Scaffolds was employed to gene prediction and annotation after with a length over 300 bp were extracted and broken into contigs without gaps. The software CD-HIT (http://www.bioinformatics.org/cd-hit/) was employed to all sequences sequence identity (90% coverage) from gene sets with ≥95%, and were clustered as the non-redundant gene catalog using. After quality control, the software SOAPaligner (http://soap.genomics.org.cn/) was employed to mapped reads to representative genes with ≥95% identity, and the gene abundances were evaluated in each sample. The software BLASTP (Version 2.2.28+, http://blast.ncbi.nlm.nih.gov/Blast.cgi) was employed to taxonomic annotations by aligning non-redundant gene catalogs against NCBI NR database with cutoff: 1e^− 5^(e-value). The software BLASTP (Version 2.2.28+) was employed to annotation the KEGG pathway search against the Kyoto Encyclopedia of Genes and Genomes database (http://www.genome.jp/keeg/) with an cutoff: 1e^− 5^ (e-value).

### Statistical analyses

Trimmomatic software was used to excise primers and for quality filtering with the original metagenomic sequences [27]. MetaPhlan2 software was then used to analyze the data and obtain the classifications for the microbial population with a degree of horizontal precision [28]. The species concentration in each sample type was calculated by comparing the mean and median relative abundances in the saline and nonsaline soil samples. R was used to conduct statistical analyses and to plot the taxonomic information at the genus level. The Shannon diversity index was calculated using the “vegan” package [29]. The Bray–Curtis dissimilarity between different sample types was calculated using the R package “ecodist” [30].

KEGG Orthology (KO) functional profiling of the soil microbiota was performed using assemblies derived from whole-genome shotgun sequencing data. Low-quality reads were first trimmed from raw sequencing data using Trimmomatic. High-quality reads were assembled de novo into contigs using metaSPAdes [31] with the default parameters. Next, we performed gene prediction for these scaffolds using PROKKA V.1.11 [32] and the predicted proteins were assigned to the KO using the Kyoto Encyclopedia of Genes and Genomes (KEGG) Automatic Annotation Server. Trimmed high-quality reads located on the given scaffolds were counted to calculate the abundances of Kos in each sample using the Burrows–Wheeler Aligner [33]. The matrix was normalized by dividing the absolute amount of each functional gene by the total number of reads assigned to functional genes in each sample in order to determine the differential expression of microbial functional pathways in the saline and nonsaline soil samples.

The bacterial correlations in the saline and nonsaline soil samples were computed based on the relative abundance of each genus using SparCC with 100 bootstraps to estimate the *p*-values for co-occurrence network analysis. The correlation values *p* < 0.05 were retained. The co-occurrence network obtained for the microbial communities in the saline and nonsaline soil samples was visualized with Gephi (version 0.9.1, https://gephi.org/). The closeness values and eigenvectors were calculated for the nodes to measure the node centralities in each network.

Community clustering was conducted based on the Bray–Curtis distance for principal coordinate analysis at the genus level, and ADONIS (“vegan”) analysis was performed to assess the similarities between groups and the significance of the differences between groups. Genera variation analysis to contrast the two soil sample types was conducted using the Wilcoxon rank sum test with R. Checks and corrections of the false discovery rate (FDR) were performed using the R program fdrtool.

## Results and discussion

### Soil characteristics of Tibetan plateau

Soil geochemical analysis showed that the EC values determined for the saline soil were about 9 ds·m^− 1^ (Fig. 2), and thus the soils were moderately saline [34]. The EC values of the nonsaline soil were less than 4 ds·m^− 1^. The pH values were ~ 4.5 for the saline soil and ~ 7.2 for the nonsaline soil. TOC of saline and nonsaline soil were about 1.2% and about 4.1% respectively. The soil moisture contents were about 7% higher in nonsaline soil than the saline soil, possibly because salinization destroyed the physical structure of the soil. The nitrate, sulfate, and TS accumulations were significantly higher in the saline soil than the nonsaline soil, whereas TN and AN were significantly lower in the saline soil. Thus, the saline soil was acidic and the nutrient nitrogen contents were lower. The long-term accumulation of heavy metal (loid) s resulted in an extremely high Mn content. The levels of heavy metal (loid) s such as Zn, As, Cu, and Cr, and metal cations such as K^+^, Ga^2+^, and Mg^2+^ were also significantly higher in saline soil than nonsaline soil (Fig. S2). In general, the saline and nonsaline soil differed significantly in terms of most of the geochemical parameters (FDR < 0.05, Fig. S2). The saline soil had a low pH, high salinity, and low nutrient levels, and it was also affected by the extreme unique climate of the Tibetan Plateau, including cold, hypoxia, and strong ultraviolet radiation. Microorganisms may produce a series of community changes and genetic selection under such environmental conditions.

### Bacterial community in saline and nonsaline soil of Tibetan plateau

**Fig. 1:**
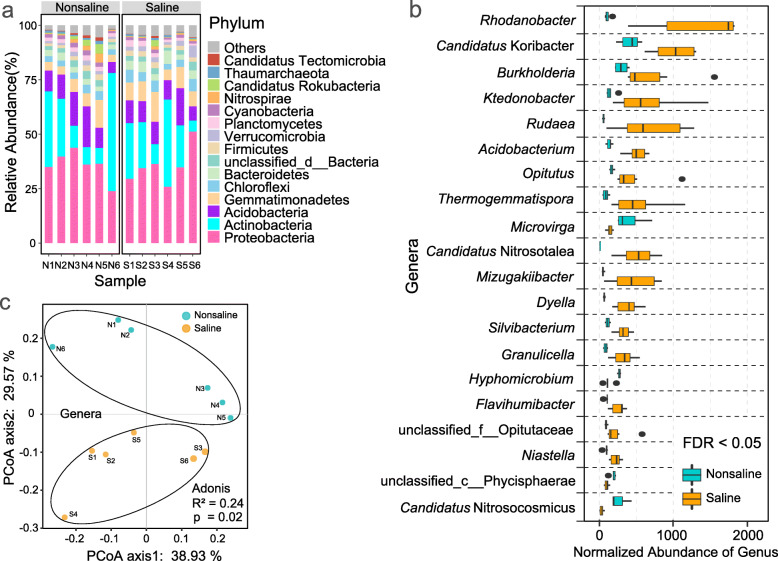
Composition and differences in saline and nonsaline soil on the Tibetan Plateau. a. Bacterial composition at the phylum level in saline and nonsaline soil samples of Tibetan Plateau. b. Top 20 genera with significant differences (FDR < 0.05) in saline (orange) and nonsaline (cyan) soil samples of Tibetan Plateau, the total number of reads is normalized to 100,000. c. Principal coordinate analysis of saline (orange) and nonsaline (cyan) soil samples based on the composition and abundances of the bacterial communities at the genus level

The Shannon index was 4.99–5.66 for saline soil and that for nonsaline soil was 5.28–5.40 (Table S1). In all samples, bacteria accounted for approximately 98.07% of the total sequences, archaea accounted for approximately 1.32%, and fungi accounted for only 0.01%. The microbial community was dominated by bacteria, and the proportions of archaea and fungi were extremely low. At the microbial phylum level, the sequences were dominated by Proteobacteria, Actinobacteria, Acidobacteria, Gemmatimonadetes, Chloroflexi, and other phyla (Fig. 1a).

By analyzing the differences in the bacterial compositions in two types of soil samples (Wilcoxon’s test, FDR < 0.05, Fig. 1b), we found that the significantly enriched bacteria in the saline soil had different metabolic strategies. Most were heterotrophic bacteria, but some were chemoautotrophs, such as *Rhodanobacter*, *Granulicella*, and *Acidobacterium*. Furthermore, *Opitutus* and *Mizugakiibacter* are capable of reducing nitrate to nitrite. *Thermogemmatispora, Silvibacterium* and *Niastella* participated in carbon cycle through degrading polysaccharides such as cellulose, starch, chitin and xylan, etc. The dominant significantly enriched bacterial groups associated in the carbon and nitrogen cycles identified in nonsaline soil. For example, *Microvirga* and *Hyphomicrobium* have denitrification functions [35, 36], and *Candidatus* Nitrosocosmicus has ammonia oxidation and carbon fixation capacities [37]. The bacteria in saline soil were found to have specific environmental (low pH, high salinity, and low nutrient levels.) adaptations (such as chemoautotrophs and obligate acidophilic), whereas the dominant bacteria in nonsaline soil have greater capacity for carbon and nitrogen assimilation. PCoA showed that all of the saline soil samples clustered together and those in nonsaline soil samples formed another cluster, thereby indicating that soil salinization led to significant differences in the bacterial community structure (Fig. 1c).

The results of the functional abundance based on the KEGG database (Fig. S3a) showed that significant differences in functional genes related to environmental stress resistance (ultraviolet radiation resistance, temperature change response, oxygen limitation response, and salinity adaptation) and important biogeochemical processes (e.g., carbon fixation, nitrogen metabolism, sulfur metabolism, methane metabolism, and heavy metal resistance). PCoA based on the functional composition showed (Fig. S3b) that two separate clusters were formed at module level, indicating that salinization also led to significant differences in the soil bacterial functions (ADONIS, *p*-value < 0.05). The diversity of composition of bacterial community in non-saline soil was positively correlated with diversity of diversity of functions, while negatively correlated in the saline soil (Fig. S3b and c). However, the certain bacterial metabolism related to different element cycles and environmental stress responses should be revealed deeply.

### Metabolic pathways in saline and nonsaline soil samples of Tibetan plateau

#### Carbon cycling

**Fig. 2:**
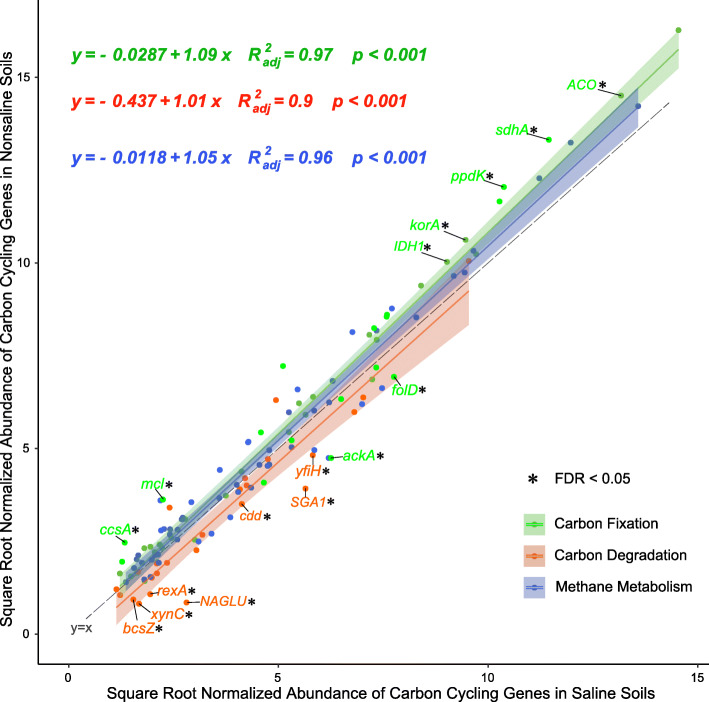
The ratio of genes in carbon cycling pathway in saline and nonsaline soil on the Tibetan Plateau. The black dotted line equation “y = x” indicates that the horizontal and vertical axes are equal. Green, red, and blue represent genes of carbon fixation, carbon degradation, and methane metabolism pathways, respectively. Genes with significant differences (FDR < 0.05) are marked in the corresponding colors and connected with short lines. The total number of reads is normalized to 100,000

The carbon cycle mainly comprises carbon fixation, carbon degradation, and methane metabolism, which is important for microorganisms in the soil to obtain energy and materials [38]. The carbon fixation pathways in all the samples were mainly composed of the reductive citrate (rTCA) cycle, hydroxypropionate hydroxybutylate (3-HP/4-HB) cycle, crassulacean acid metabolism (CAM) pathway, and Wood–Ljungdahl (WL) pathway (Fig. S4a). In Fig. 2, the genes such as *korA*, *sdhA* and *ppdK* with significantly higher abundances in saline soil participated in the 3-HP/4-HB cycle and WL pathway, whereas the genes with significantly higher abundances in nonsaline soil mainly participated in the rTCA cycle. The rTCA cycle is prevalent in anaerobic bacteria that are adapted to hypoxic environments on the Tibetan Plateau and this cycle only requires two ATP equivalents to form pyruvate [39]. The abundance of the WL pathway was probably higher because of its extremely low energy consumption (requirement < 1 ATP) and requirement for strict anoxic conditions [40]. Thus, low nutritional availability and extreme environments explain why the rTCA cycle and WL pathway predominate in the soil on the Tibetan Plateau, where the 3-HP/4-HB cycle pathway are adaptations to the nutritional deficiencies, respectively.

In the carbon degradation pathway (Fig. 2), the genes with significantly higher abundances in saline soil included genes related to starch degradation comprising *cdd* and *SGA1*, chitin degradation gene *NAGLU*, lignin degradation gene *yfiH*, cellulose degradation gene *bcsZ*, hemicellulose degradation gene *xynC*, and *rexA*, indicating that the potential for carbon degradation was greater in the saline soil. Interestingly, the abundances of genes associated with the degradation of labile carbon (starch, pectin, and hemicellulose) and recalcitrant carbon (cellulose, chitin, and lignin) were higher in the saline soil, possibly because these mechanisms allow microorganisms to maintain their ecosystem functions in the short term in saline soil. In addition, these mechanisms may also explain why the TOC contents were significantly lower in saline soil than nonsaline soil, and the collapse of farmland ecosystems may occur if saline soil remains oligotrophic for a long time [21, 22]. There were no significant differences in the abundances of genes related to methane metabolism in the two soil types (Fig. 2 and Fig. S4b). The lack of significant differences in abundances of genes related to this process indicates that salinization of the soil had no significant impacts on methane metabolism.

#### Nitrogen cycling

**Fig. 3:**
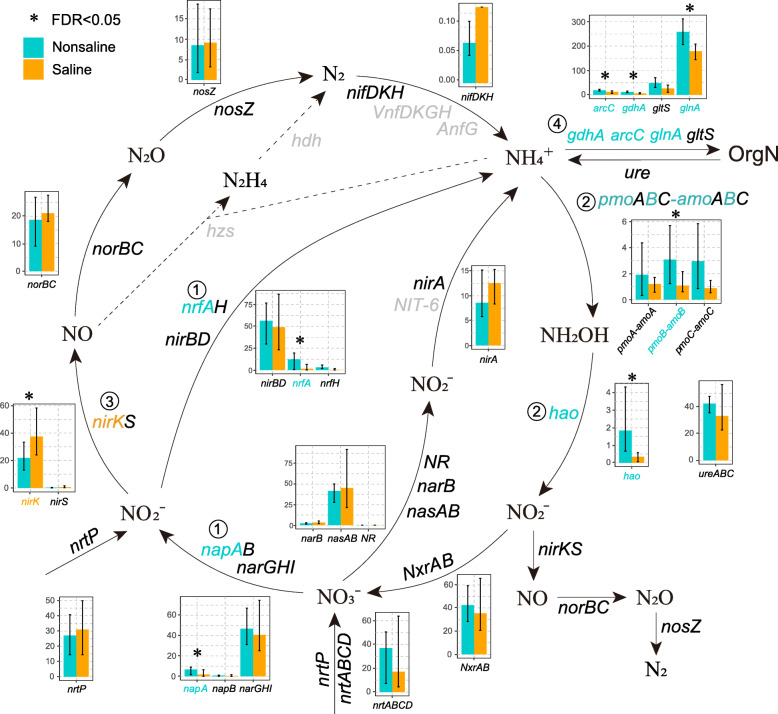
Differences in the abundance of genes related to nitrogen cycling in the saline (orange) and nonsaline (cyan) soil on the Tibetan Plateau. Bar plots show the normalized abundances of nitrogen cycling genes. Significantly different (FDR < 0.05) genes are marked with “*” and circled numbers. Undetected genes are indicated in gray. Circled numbers identify genes with significant differences: 1, dissimilatory nitrate reduction; 2, nitrification; 3, denitrification; and 4, organic nitrogen conversion. The total number of reads is normalized to 100,000

The nitrogen cycle is one of the crucial soil nutrient cycle processes for the growth of crops and it is driven by soil microorganisms with specific functions [41]. The abundances of genes related to dissimilatory nitrate reduction and denitrification in the soil microbial nitrogen cycle differed significantly in saline and nonsaline soil (Fig. 3), but they did not differ significantly in the nitrate assimilation reduction pathway.

In the denitrification pathway, the abundance of gene *nirK* (nitrite reductase catalyzing N_2_O to NO) was significantly higher in saline soil (FDR < 0.05) than nonsaline soil. The presence of higher amounts of NO is likely to proceed forward to produce more N_2_O. In saline soil, bacteria have the potential to produce more NO and N_2_O via denitrification, and the abundances of genes *nosZ* that could reduce N_2_O were lower in saline soil than nonsaline soil, which were resulted in more NO and N_2_O produced in saline soil. The abundances of the dissimilatory nitrate reduction genes *napA* and *nrfA* were significantly higher (FDR < 0.05) in saline soil than nonsaline soil (Fig. 3), demonstrating that the potential for ammonia conversion was higher in nonsaline soil. Significantly higher abundances were found in nonsaline soil of the gene *gdhA* encoding the enzyme that catalyzes the conversion of ammonia to L-glutamate, which are all involved in the conversion of ammonia to glutamic acid (FDR < 0.05). It is indicated that more ammonia could be converted into organic nitrogen and this is beneficial for the production of crops. Microorganisms need to synthesize large amounts of amino acids to resist environmental pressure [42]. Therefore, the input of ammonia is critical for the microbial community.

#### Sulfur metabolism

**Fig. 4:**
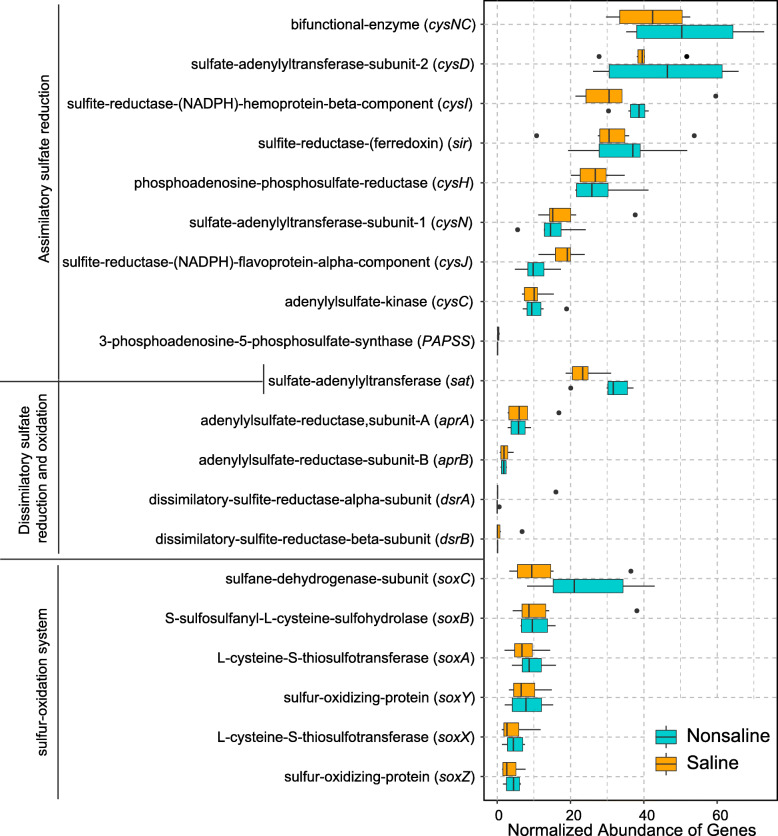
Difference in abundances of sulfur cycling genes in saline (orange) and nonsaline (cyan) soil on the Tibetan Plateau. The total number of reads is normalized to 100,000

Due to the high input of sulfate in saline soil, we analyzed the differences in the genes abundance of three pathways related to sulfur metabolism (assimilatory sulfate reduction, dissimilatory sulfate reduction and oxidation, and SOX system) (Fig. 4). The abundances of genes related to environmental sulfide absorption were significantly lower in saline soil, but the abundances of genes associated with the elimination of toxic intracellular sulfide were significantly higher.

First, in saline and nonsaline soil, the abundance was high for the assimilatory sulfate reduction module that consumes sulfate in the environment and ultimately synthesizes sulfur-containing amino acids. This pathway was probably dominant because of the extremely high sulfate content in saline soil and the large demand for amino acids in the bacterial community. Second, in both soil types, the abundance was low for the dissimilatory sulfate reduction and oxidation module that produces energy and inorganic sulfides, and the abundance of *dsrAB* (dissimilatory sulfite reductase) was extremely low, probably because “reverse” sulfite reductase (*dsr*) is not necessary for the oxidation of sulfide or thiosulfate, and *sat* (encoding the enzyme that catalyzes the conversion of sulfate to Adenylyl sulfate) and *aprAB* (adenylylsulfate reductase) participate in the energy production process. Finally, the abundance of the *soeBC* gene encoding the enzyme in the SOX system that catalyzes the conversion of sulfite into sulfate was significantly higher in saline soil, and this enzyme is important for sulfite oxidation in the cytoplasm (Fig. S5). The oxidation of sulfur can reduce the toxicity of sulfite in cells [43], as well as providing electrons and energy to cells [44].

#### Metal resistance

**Fig. 5:**
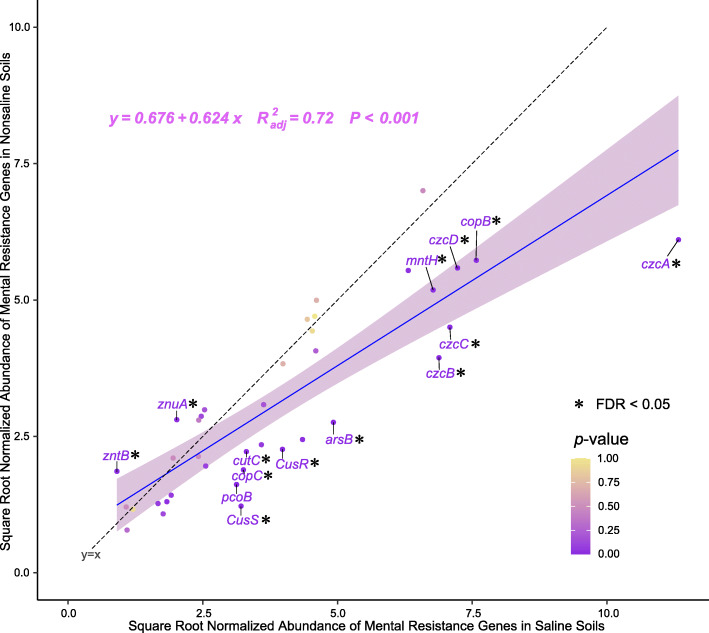
Abundance of heavy metal (loid) resistance genes in saline and nonsaline soil on the Tibetan Plateau. The black dotted line with the equation “y = x” indicates that the horizontal and vertical axes are equal. Genes with significantly different abundances (FDR < 0.05) are marked in purple and connected with short lines. The total number of reads is normalized to 100,000

In this study, the abundances of heavy metal (loid) resistance genes such as *copB*, *cutC*, *cusRS*, and *pcoB* genes that confer tolerance to copper and the manganese transport gene *mntH* were significantly higher in saline soil (Fig. 5). The differences in the functional genes related to heavy metal absorption showed that the gene *arsB* related to arsenic absorption had a significantly higher abundance in saline soil (Fig. 5). Both soil types had high abundances of the arsenate reductase gene (*arsC*), but the abundance of the arsenite oxidase gene (*aoxAB*) was extremely low because the hypoxic environment promoted the migration of arsenic [45]. The accumulation of arsenite in saline soil on the Tibetan Plateau has toxic effects on microorganisms and crops. However, the abundance of gene *arsH* (arsenical resistance protein) was extremely low in both soil types (Fig. S6), and thus, the soil bacteria did not have a high capacity to resist the accumulation of arsenic in study area.

These results indicate that two main mechanisms mediate microbial resistance to the toxicity of heavy metal (loid) s in saline soils on the Tibetan Plateau, i.e., the efflux of excessive concentrations of heavy metal (loid) ions from cells and expressing proteins that confer tolerance of heavy metal (loid) ions. However, bacteria to resist increasing concentrations of heavy metal (loid) s is possibly not sufficient for increasing concentration of heavy metal (loid) ions in the soil of Tibetan Plateau, which should be studied in future research.

#### Environmental stress response

**Fig. 6:**
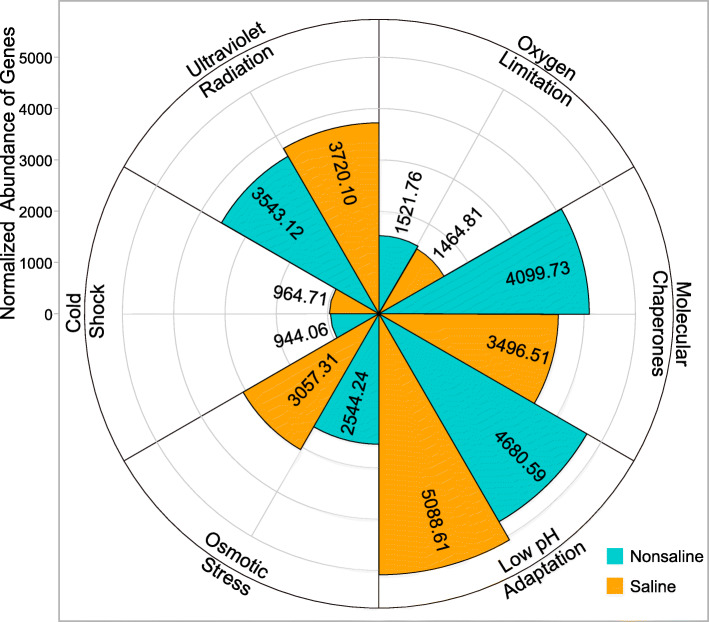
Abundance of genes associated with the environmental stress resistance. The total number of reads is normalized to 100,000

Ultraviolet radiation resistance, the nucleotide excision repair pathway, and photoreactivation (DNA repair via the photolysis enzyme encoded by the *phrB* gene) are applied by bacteria to avoid and repair damage due to ultraviolet radiation [46]. The abundances of the genes such as *recNO* and *alkB* associated with DNA repair were significantly higher in saline soil due to more DNA damage in saline soil (Fig. 6 and Fig. S8). Bacteria adapt to cold environments mainly through cold shock genes *cspA*, *desK*, and *desR*, which encode enzymes that protect cells from ice crystal damage, and that maintain the transcription and translation processes within cells [47, 48], and via lipid desaturases (*desA1*, *desA2*, and *desC*; Fig. S7) and the synergy among unsaturated fatty acid synthesis genes *fabAB* (anaerobic) and *desAB* (aerobic), which are responsible for synthesizing short-chain unsaturated fatty acids embedded in the cell membrane to maintain the cell membrane fluidity and avoid film hardening at low temperatures [49, 50]. These genes were abundant in the two soil types on Tibetan Plateau (Fig. S7). Bacteria express large amounts of catalase and peroxidase when responding to oxygen limitation stress [37]. Thus, the abundances of the *cydB*, *fnr*, and *oxyR* genes were significantly higher in saline soil (Fig. S7), whereas the *katE* (catalase) gene helping cells cope with environmental pressures was more abundant in nonsaline soil. The response mechanisms to oxygen limitation possibly differed between the two soil types. Molecular chaperones help protein folding and refolding to enhance stress resistance [51], and many of these genes had high abundances in the two sample types (Fig. S7). However, the abundances of *danK* and *groEL* were significantly (FDR < 0.05) higher in saline soil, whereas *grpE* and *pccA* were more abundant in nonsaline soil, and the abundances of major molecular chaperone genes were higher in nonsaline soil than saline soil, which lacked sufficient energy and substrates to synthesize the required molecular chaperones (Fig. S7).

In response to high salinity and low pH, the abundance of the K^+^ high-affinity transport system (*kdpABC*) was significantly higher in saline soil (Fig. S7). Bacteria generate an inward positive membrane potential through the active inflow of K^+^ to partially deflect the inward flow of protons [52], as well as helping cells to resist the stress due to high osmotic pressure. The metabolism of proton buffer molecules can also maintain the pH in the cytoplasm, and the abundance of the phosphate uptake gene *pstS* was significantly higher in saline soil (Fig. S7). Cross-protective genes encoding proteins (*osmC*, *dps*, and *katE*) that maintain the normal life activities of cells under high osmotic pressure were abundant in saline soil. In addition, the microbial self-synthesizing glycine betaine gene *gbsAB* was more abundant in nonsaline soil, whereas proline and glycine betaine absorption genes (*opuABCD*, *proP*, *putABP*, etc.) were more abundant in saline soil (Fig. S7), probably because the energy consumed by the synthetic permeate was higher than that absorbed from the environment. Therefore, the soil bacteria in saline soil were deficient in substances and energy, so they employed low energy consumption mechanisms to absorb K^+^, phosphate, and osmotic substances from the environment, as well as synthesizing a small amount of protective proteins to resist the low pH and high osmotic pressure stresses.

Thus, the bacterial community in saline soil was affected by more extreme environmental stresses than that in nonsaline soil. Low pH and high osmotic pressure resistance genes were more abundant, and the abundances of molecular chaperone genes were significantly lower. Genes related to adaptability to the specific climatic conditions on the Tibetan Plateau were abundant and the differences in their abundances of two soil types were not obvious.

#### Effects of physicochemical parameters on microbial community and metabolic capacity

**Fig. 7:**
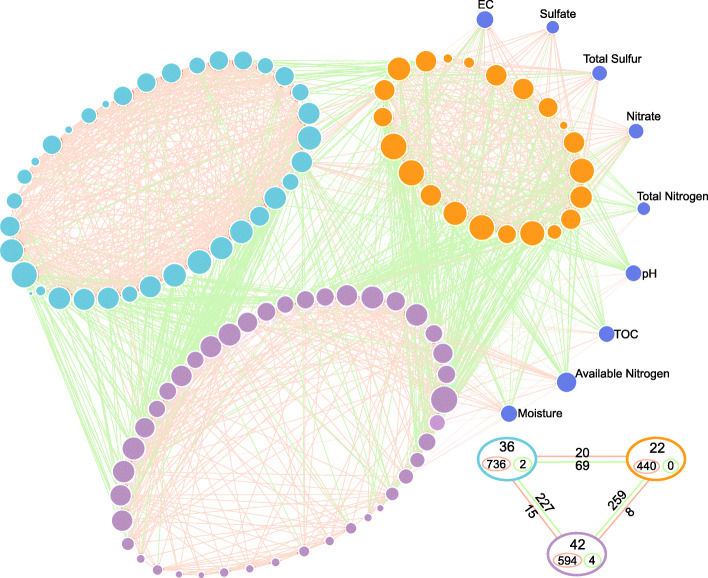
Network of the top 100 dominant genera and physicochemical parameters. A connection denotes a Spearman’s correlation coefficient with a magnitude greater than 0.6 (positive correlation = red lines) or less than − 0.6 (negative correlation = green lines) and statistically significant (*p*-value < 0.05). The size of each node is proportional to the number of connections (i.e., degree). The thickness of each connection between two nodes (i.e., edge) is proportional to the Spearman’s correlation coefficient (i.e., weight), ranging from |0.6| to |1|. The nodes clustered in the same module share the same color. The lower right corner is a schematic diagram, and the large circles represent three modules based on network analysis, and the positions are corresponding. In the large circles, the top number indicates the number of nodes, the red circle and the number indicate positive correlation within the module, and the green indicates negative correlation. Between the large circles, red lines and numbers indicate positive correlations, and green lines and numbers indicate negative correlations

Co-occurrence network analysis based on the bacterial genera, functional genes, and environmental factors was constructed in order to understand how environmental factors affect the complex bacterial community structure and functions in saline and nonsaline soils on the Tibetan Plateau (Fig. 7 and S8). The network based on the bacterial community and environmental factors contained three bacterial modules (Table S2). The main negative correlations were found between these three modules, but the bacterial genera within the same module were mainly positively correlated (Fig. 7).

Module 0 (Orange) contained a total of 22 nodes and most of them (> 50%) represented microbial genera with a significant (FDR < 0.05) higher abundance in saline soil. Most of the genera in this module are chemoautotrophic and acidophilic bacteria (Table S3 and Fig. 1a), which have strongest correlations with the soil physicochemical parameters, including positive correlations with EC, sulfate, total sulfur, and nitrate, but mostly negative correlations with pH, TN, AN moisture, and TOC (Fig. 7). Module 1 (purple) contained 42 nodes, and all of the genera are high abundant in the two soil types. These genera had strong positive correlations with the soil moisture, TOC, and AN, weak positive correlations with TN and pH (Fig. 7). Module 2 (cyan, 36 nodes) had very weak negative correlations with TN and pH. Bacterial community responded to the environmental factors by forming different functional groups (Fig. 7), indicating that the stress due to salinity indeed altered the topological roles of microbes and reorganized the keystone populations [53].

The correlation between the non-saline soil bacterial community and soil properties is more significant than saline soil (Fig. S8a). The bacterial community in saline soil is more significantly affected by properties such as EC, pH, AN, Mn, and Zn. Based on the mental test, we established a network of functional genes and environmental factors. It showed that the main physical and chemical parameters in saline soil (EC, pH, TOC, moisture, AN, TS, etc.) affected the key biogeochemical cycles for C, N, S, As, and other elements in the soil (Fig. S8b). TOC was significantly positively correlated with rTCA cycling which was a high energy efficiency conversion rate pathway in the carbon cycle. However, TOC was significantly negatively correlated with the WL pathway, which may have been related to the extremely low energy consumption and strict anaerobic requirements of this pathway. In addition, TOC, moisture, and pH were significantly negatively correlated with the main carbon degradation genes (*cdd*, *SGA1*, *rexA*, *xynC*, *bcsZ*, and *NAGLU*), and the high abundance of carbon degradation genes resulted in a significant decrease in the soil TOC concentration (Fig. 3). EC was negatively correlated with carbon fixation genes and positively correlated with carbon degradation genes, indicating that high salinity led to the rapid degradation of organic carbon for the bacterial requirements for substrates and energy. Most of the genes in nitrogen cycle were also positively correlated with TN, AN moisture, and pH, but negatively correlated with EC (Fig. S8b). In particular, the abundance of gene *nirK* was significantly negatively correlated with TN, but significantly positively correlated with pH, indicating that the denitrification process in saline soil was affected by changes in the soil physicochemical properties, and the long-term accumulation of salinity may have led to the accumulation of nitrate and enhanced denitrification. In addition, sulfate and total sulfur were significantly negatively correlated with the sulfate absorption gene *cysAPUW*, but significantly positively correlated with the sulfur oxidation gene *soeABC*, while the key gene *sat* in the sulfate reduction pathway was significantly negatively correlated with EC. These results suggest that the accumulation of sulfide in saline have resulted in the bacteria producing more sulfur oxidation genes to synthesize more sulfate. The accumulation of salt would happen in the soil. Moreover, the soil moisture and pH were negatively correlated with arsenic resistance genes, whereas EC had positive correlations. Overall, high salinity had significant impacts on the microbial community and its metabolic network, with significant increases in the chemoautotrophic and acidophilic bacterial modules, as well as effects on other heterotrophic bacteria modules related to the carbon and nitrogen cycles. The bacterial community in the saline soil is likely to consume more soil organic carbon to increase denitrification and intensify the oxidation of sulfur.

## Conclusions

Global warming has caused the melting of glaciers and the repeated freezing and thawing of the permanently frozen soil on the Tibetan Plateau, resulting in a sudden increase in the water volume and the exposure of rock formations, where the water flow has washed over these mineral-rich rock formations into rivers to subsequently increase the soil salinity via irrigation. In this study, we found that the salinization of the soil was accompanied by increased acidity and the accumulation of metal. People who live along the rivers on the Tibetan Plateau are affected by the risk of increasingly saline farming soil. Soil salinization significantly changed the bacterial communities on the Tibetan Plateau, and chemoautotrophic and acidophilic bacteria became dominant. In addition, the key biogeochemical cycle function clusters changed. Carbon degradation, denitrification, and sulfur oxidation gene clusters were highly abundant in saline soils, which were associated with the loss of soil organic matter, increased emissions of NO and N_2_O, and higher sulfate levels in local area. The bacterial community adapting to saline soil did not alleviate the degree of soil salinity, and bacterial community is likely to consume more energy to cope with the extreme climate under saline soil conditions due to the unique features of the Tibetan Plateau. Soil salinization also becomes one of the most direct forms of feedback on the Tibetan Plateau in response to global climate change.

## Supplementary Information

**Additional file 1: Figure S1.** Sampling sites on the Tibetan Plateau. **Figure S2.** Physicochemical parameters in saline and nonsaline soil on the Tibetan Plateau. **Figure S3.** The difference of bacterial community functions in saline and nonsaline soil on the Tibetan Plateau. **Figure S4.** Normalized abundance of functional pathways of carbon fixation (a) and methane metabolism (b) of bacterial community in saline and nonsaline soil on the Tibetan Plateau. **Figure S5.** The ratio of sulfur cycling genes in saline and nonsaline soil on the Tibetan Plateau. **Figure S6.** The normalized abundance of heavy metal(loid)s resistance genes in saline and nonsaline soil on the Tibetan Plateau. **Figure S7.** The normalized abundance of environmental stress response genes in saline and nonsaline soil on the Tibetan Plateau. **Figure S8.** The correlation analysis of bacterial community composition and function with environmental factors in saline and nonsaline soil on the Tibetan Plateau. **Table S1.** The alpha diversity of bacterial community in saline and nonsaline soils of Tibetan Plateau. **Table S2.** The topology structure characteristics of network of bacteria and environmental factors. **Table S3.** Genera in different modules in network of genus and environmental factors.

## Data Availability

The raw sequence data reported in this paper have been deposited in the Genome Sequence Archive (Genomics, Proteomics & Bioinformatics 2017) in National Genomics Data Center (Nucleic Acids Res 2021), China National Center for Bioinformation / Beijing Institute of Genomics, Chinese Academy of Sciences, under accession number CRA003771 that are publicly accessible at (http://bigd.big.ac.cn/gsa/s/6AYB1I5n).

## References

[1] Kang S, Xu Y, You Q, Flügel W-A, Pepin N, Yao T (2010). Review of climate and cryospheric change in the Tibetan Plateau. Environ Res Lett.

[2] Guan X, Wang J, Zhao H, Wang J, Luo X, Liu F, Zhao F (2013). Soil bacterial communities shaped by geochemical factors and land use in a less-explored area, Tibetan plateau. BMC Genomics.

[3] Qiu J (2008). China: the third pole. Nature.

[4] Zhang H, Fan J, Wang J, Cao W, Harris W (2018). Spatial and temporal variability of grassland yield and its response to climate change and anthropogenic activities on the Tibetan plateau from 1988 to 2013. Ecol Indic.

[5] Cheng G, Wu T (2007). Responses of permafrost to climate change and their environmental significance, Qinghai-Tibet Plateau. J Geophysical Res.

[6] Luo S, Wang S, Tian L, Shi S, Xu S, Yang F, Li X, Wang Z, Tian C (2018). Aggregate-related changes in soil microbial communities under different ameliorant applications in saline-sodic soils. Geoderma.

[7] Che R, Wang Y, Li K, Xu Z, Hu J, Wang F, Rui Y, Li L, Pang Z, Cui X (2019). Degraded patch formation significantly changed microbial community composition in alpine meadow soils. Soil Tillage Res.

[8] Li HY, Webster R, Shi Z (2015). Mapping soil salinity in the Yangtze delta: REML and universal kriging (E-BLUP) revisited. Geoderma.

[9] Csonka LN (1989). Physiological and genetic responses of bacteria to osmotic stress. Microbiol Mol Biol Rev.

[10] Zhou M-X, Renard M-E, Quinet M, Lutts S (2019). Effect of NaCl on proline and glycinebetaine metabolism in Kosteletzkya pentacarpos exposed to cd and Zn toxicities. Plant Soil.

[11] Asghar HN, Setia R, Marschner P (2012). Community composition and activity of microbes from saline soils and non-saline soils respond similarly to changes in salinity. Soil Biol Biochem.

[12] Zheng W, Xue D, Li X, Deng Y, Rui J, Feng K (2017). Wang Z-l: the responses and adaptations of microbial communities to salinity in farmland soils: a molecular ecological network analysis. Appl Soil Ecol.

[13] Zhang K, Shi Y, Cui X, Yue P, Li K, Liu X, Tripathi BM, Chu H. Salinity is a key determinant for soil microbial communities in a desert ecosystem. mSystems. 2019;4(1):e00225–18. 10.1128/mSystems.00225-18. 10.1128/mSystems.00225-18 PMC6372838 30801023

[14] Yu Y, Zhao C, Zheng N, Jia H, Yao H (2019). Interactive effects of soil texture and salinity on nitrous oxide emissions following crop residue amendment. Geoderma.

[15] Ma L, Ma F, Li J, Gu Q, Yang S, Wu D, Feng J, Ding J (2017). Characterizing and modeling regional-scale variations in soil salinity in the arid oasis of Tarim Basin, China. Geoderma.

[16] Khan KS, Mack R, Castillo X, Kaiser M, Joergensen RG (2016). Microbial biomass, fungal and bacterial residues, and their relationships to the soil organic matter C/N/P/S ratios. Geoderma.

[17] Rath KM, Maheshwari A, Rousk J (2017). The impact of salinity on the microbial response to drying and rewetting in soil. Soil Biol Biochem.

[18] Wichern J, Wichern F, Joergensen RG (2006). Impact of salinity on soil microbial communities and the decomposition of maize in acidic soils. Geoderma.

[19] Yan N, Marschner P, Cao W, Zuo C, Qin W (2015). Influence of salinity and water content on soil microorganisms. Int Soil Water Conserv Res.

[20] Li X, Sun M, Zhang H, Xu N, Sun G (2016). Use of mulberry-soybean intercropping in salt-alkali soil impacts the diversity of the soil bacterial community. Microb Biotechnol.

[21] Chu H, Wang S, Yue H, Lin Q, Hu Y, Li X, Zhou J, Yang Y (2014). Contrasting soil microbial community functional structures in two major landscapes of the T ibetan alpine meadow. Microbiol Open.

[22] Qi Q, Zhao M, Wang S, Ma X, Wang Y, Gao Y, Lin Q, Li X, Gu B, Li G (2017). The biogeographic pattern of microbial functional genes along an altitudinal gradient of the Tibetan pasture. Front Microbiol.

[23] Chen FH, Dong GH, Zhang DJ, Liu XY, Jia X, An C-B, Ma MM, Xie YW, Barton L, Ren X (2015). Agriculture facilitated permanent human occupation of the Tibetan plateau after 3600 BP. Science.

[24] Fan R, Short MD, Zeng S-J, Qian G, Li J, Schumann RC, Kawashima N, Smart RSC, Gerson AR (2017). The formation of silicate-stabilized passivating layers on pyrite for reduced acid rock drainage. Environ Sci Technol.

[25] Nordstrom DK (2009). Acid rock drainage and climate change. J Geochem Explor.

[26] Weatherill JJ, Krause S, Ullah S, Cassidy NJ, Levy A, Drijfhout FP, Rivett MO (2019). Revealing chlorinated ethene transformation hotspots in a nitrate-impacted hyporheic zone. Water Res.

[27] Bolger AM, Lohse M, Usadel B (2014). Trimmomatic: a flexible trimmer for Illumina sequence data. Bioinformatics.

[28] Truong DT, Franzosa EA, Tickle TL, Scholz M, Weingart G, Pasolli E, Tett A, Huttenhower C, Segata N (2015). MetaPhlAn2 for enhanced metagenomic taxonomic profiling. Nat Methods.

[29] Oksanen J, Kindt R, Legendre P, O’Hara B, Stevens MHH, Oksanen MJ, Suggests M (2007). The vegan package. Commun Ecol Package.

[30] Goslee SC, Urban DL (2007). The ecodist package for dissimilarity-based analysis of ecological data. J Stat Softw.

[31] Nurk S, Meleshko D, Korobeynikov A, Pevzner PA (2017). metaSPAdes: a new versatile metagenomic assembler. Genome Res.

[32] Seemann T (2014). Prokka: rapid prokaryotic genome annotation. Bioinformatics.

[33] Li H, Durbin R (2010). Fast and accurate long-read alignment with burrows–wheeler transform. Bioinformatics.

[34] Khorsandi F, Yazdi FA (2011). Estimation of saturated paste extracts’ electrical conductivity from 1:5 soil/water suspension and gypsum. Commun Soil Sci Plant Anal.

[35] Kanso S, Patel BK (2003). Microvirga subterranea gen. Nov., sp. nov., a moderate thermophile from a deep subsurface Australian thermal aquifer. Int J Syst Evol Microbiol.

[36] Martineau C, Mauffrey F, Villemur R (2015). Comparative analysis of denitrifying activities of Hyphomicrobium nitrativorans, Hyphomicrobium denitrificans, and Hyphomicrobium zavarzinii. Appl Environ Microbiol.

[37] Sauder LA, Albertsen M, Engel K, Schwarz J, Nielsen PH, Wagner M, Neufeld JD (2017). Cultivation and characterization of Candidatus Nitrosocosmicus exaquare, an ammonia-oxidizing archaeon from a municipal wastewater treatment system. ISME J.

[38] Shi Y, Delgado-Baquerizo M, Li Y, Yang Y, Zhu Y-G, Peñuelas J, Chu H (2020). Abundance of kinless hubs within soil microbial networks are associated with high functional potential in agricultural ecosystems. Environ Int.

[39] Hügler M, Sievert SM (2011). Beyond the Calvin cycle: autotrophic carbon fixation in the ocean. Annu Rev Mar Sci.

[40] Bar-Even A, Noor E, Milo R (2012). A survey of carbon fixation pathways through a quantitative lens. J Exp Bot.

[41] Crecchio C, Gelsomino A, Ambrosoli R, Minati JL, Ruggiero P (2004). Functional and molecular responses of soil microbial communities under differing soil management practices. Soil Biol Biochem.

[42] Tyson GW, Chapman J, Hugenholtz P, Allen EE, Ram RJ, Richardson PM, Solovyev VV, Rubin EM, Rokhsar DS, Banfield JF (2004). Community structure and metabolism through reconstruction of microbial genomes from the environment. Nature.

[43] Dahl C, Franz B, Hensen D, Kesselheim A, Zigann R (2013). Sulfite oxidation in the purple sulfur bacterium Allochromatium vinosum: identification of SoeABC as a major player and relevance of SoxYZ in the process. Microbiology.

[44] Pott AS, Dahl C (1998). Sirohaem sulfite reductase and other proteins encoded by genes at the dsr locus of Chromatium vinosum are involved in the oxidation of intracellular sulfur. Microbiology.

[45] Zhu Y-G, Williams PN, Meharg AA (2008). Exposure to inorganic arsenic from rice: a global health issue?. Environ Pollut.

[46] Thoma F (1999). Light and dark in chromatin repair: repair of UVl health iss lesions by photolyase and nucleotide excision repair. EMBO J.

[47] Ermolenko D, Makhatadze G (2002). Bacterial cold-shock proteins. Cell Mol Life Sci.

[48] Morgan-Kiss RM, Priscu JC, Pocock T, Gudynaite-Savitch L, Huner NP (2006). Adaptation and acclimation of photosynthetic microorganisms to permanently cold environments. Microbiol Mol Biol Rev.

[49] Fujii DK, Fulco AJ. Biosynthesis of unsaturated fatty acides by bacilli. Hyperinduction and modulation of desaturase synthesis. [*Bacillus megaterium*]. J Biol Chem. 1977;252(11). 405386

[50] Zhu K, Choi KH, Schweizer HP, Rock CO, Zhang YM (2006). Two aerobic pathways for the formation of unsaturated fatty acids in Pseudomonas aeruginosa. Mol Microbiol.

[51] Chen LX, Hu M, Huang LN, Hua ZS, Kuang JL, Li SJ, Shu WS (2015). Comparative metagenomic and metatranscriptomic analyses of microbial communities in acid mine drainage. ISME J.

[52] Baker-Austin C, Dopson M (2007). Life in acid: pH homeostasis in acidophiles. Trends Microbiol.

[53] Wang M, Chen S, Chen L, Wang D (2019). Responses of soil microbial communities and their network interactions to saline-alkaline stress in cd-contaminated soils. Environ Pollut.

