# Supplementary material for: Bacterial community in saline farmland soil on the Tibetan plateau: responding to salinization while resisting extreme environments
Source: BMC Microbiol. 2021 Apr 20;21:119. doi: 10.1186/s12866-021-02190-6 (PMC8056723; doi:10.1186/s12866-021-02190-6)
Supplement: Supplementary file 1 — Additional file 1: Figure S1. Sampling sites on the Tibetan Plateau. Figure S2. Physicochemical parameters in saline and nonsaline soil on the Tibetan Plateau. Figure S3. The difference of bacterial community functions in saline and nonsaline soil on the Tibetan Plateau. Figure S4. Normalized abundance of functional pathways of carbon fixation (a) and methane metabolism (b) of bacterial community in saline and nonsaline soil on the Tibetan Plateau. Figure S5. The ratio of sulfur cycling genes in saline and nonsaline soil on the Tibetan Plateau. Figure S6. The normalized abundance of heavy metal(loid)s resistance genes in saline and nonsaline soil on the Tibetan Plateau. Figure S7. The normalized abundance of environmental stress response genes in saline and nonsaline soil on the Tibetan Plateau. Figure S8. The correlation analysis of bacterial community composition and function with environmental factors in saline and nonsaline soil on the Tibetan Plateau. Table S1. The alpha diversity of bacterial community in saline and nonsaline soils of Tibetan Plateau. Table S2. The topology structure characteristics of network of bacteria and environmental factors. Table S3. Genera in different modules in network of genus and environmental factors. [file 12866_2021_2190_MOESM1_ESM.docx]

**Bacterial community in saline farmland soil on the Tibetan Plateau: Responding to salinization while resisting extreme environments**

YiQiang Li^1^, YingHui Chai^1,2^, XuSheng Wang^1^, LiYing Huang^3^, XiMing Luo^1,4^, Cheng Qiu^3^, QingHai Liu^3^, XiangYu Guan^1,4,*^

^1^ School of Ocean Sciences, China University of Geosciences (Beijing), Beijing, 100083, China

^2^ Laboratory division, Eighth Medical Center of Chinese People's Liberation Army General Hospital, Beijing100000, PR China

^3^ Institute of Agricultural Quality Standards and Testing, Tibet Academy of Agriculture and Animal Husbandry Sciences,850000, China

^4^ Beijing Key Laboratory of Water Resources and Environmental Engineering, China University of Geosciences (Beijing), Beijing, 100083, China

^*^ Corresponding author: School of Ocean Sciences, China University of Geosciences (Beijing), Beijing, 100083, China. E-mail address: guanxy@cugb.edu.cn (X. Guan).

# Supplementary Materials

**
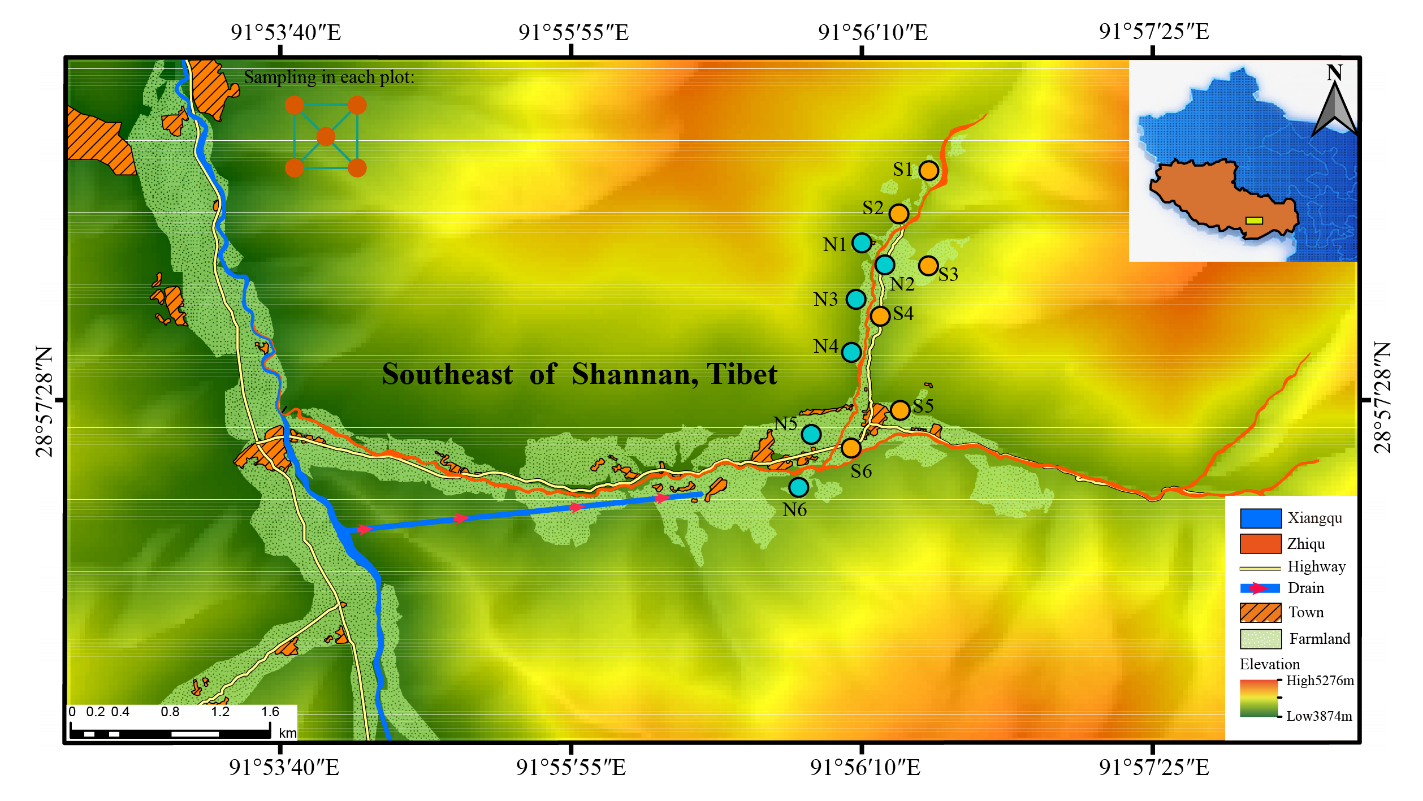
**

**Figure S1. Sampling sites on the Tibetan Plateau.**

The orange dots represent sampling sites of saline soil, and cyan dots represent sampling sites of nonsaline soil.


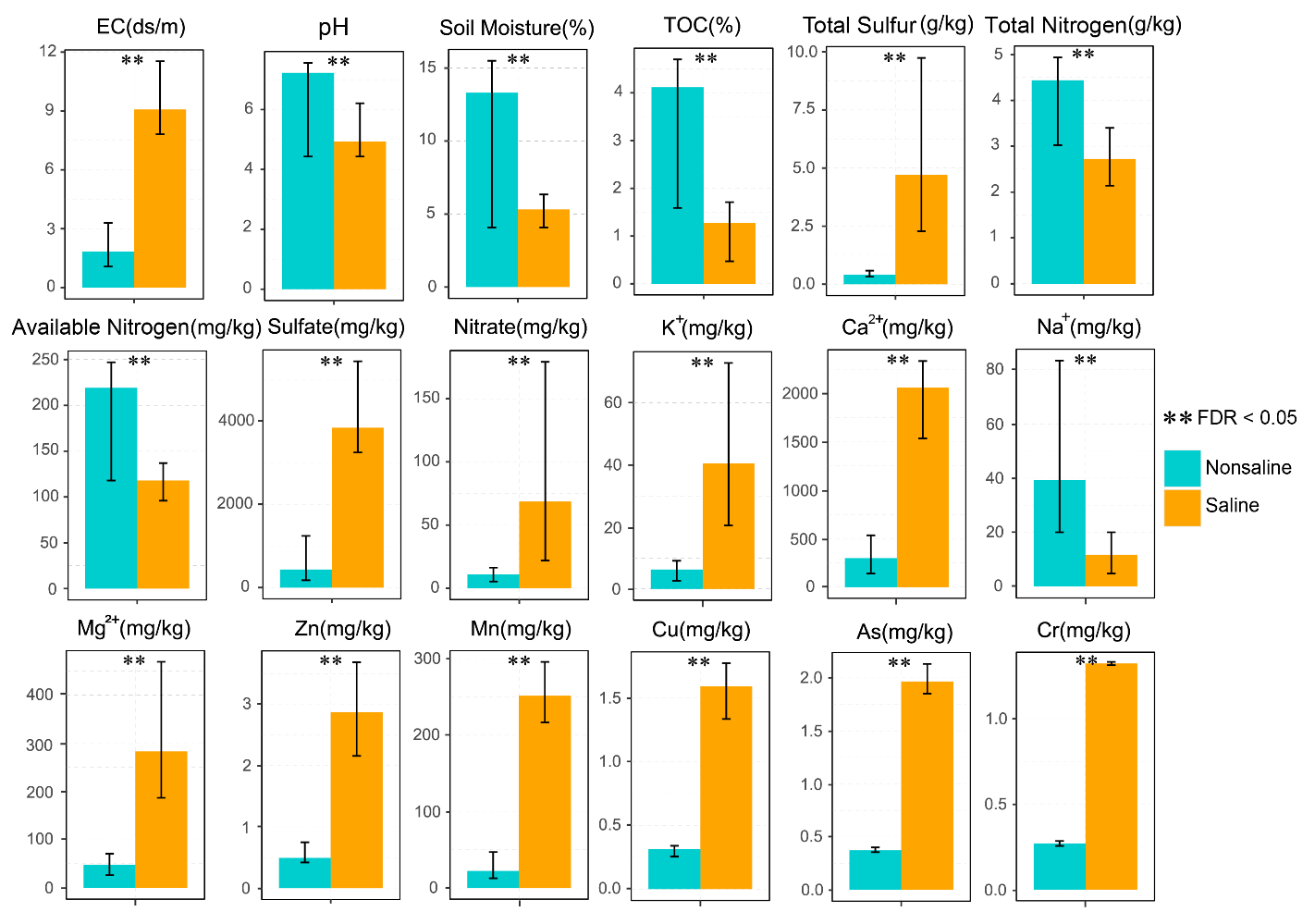


**Figure S2. Physicochemical parameters in saline and nonsaline soil on the Tibetan Plateau.**


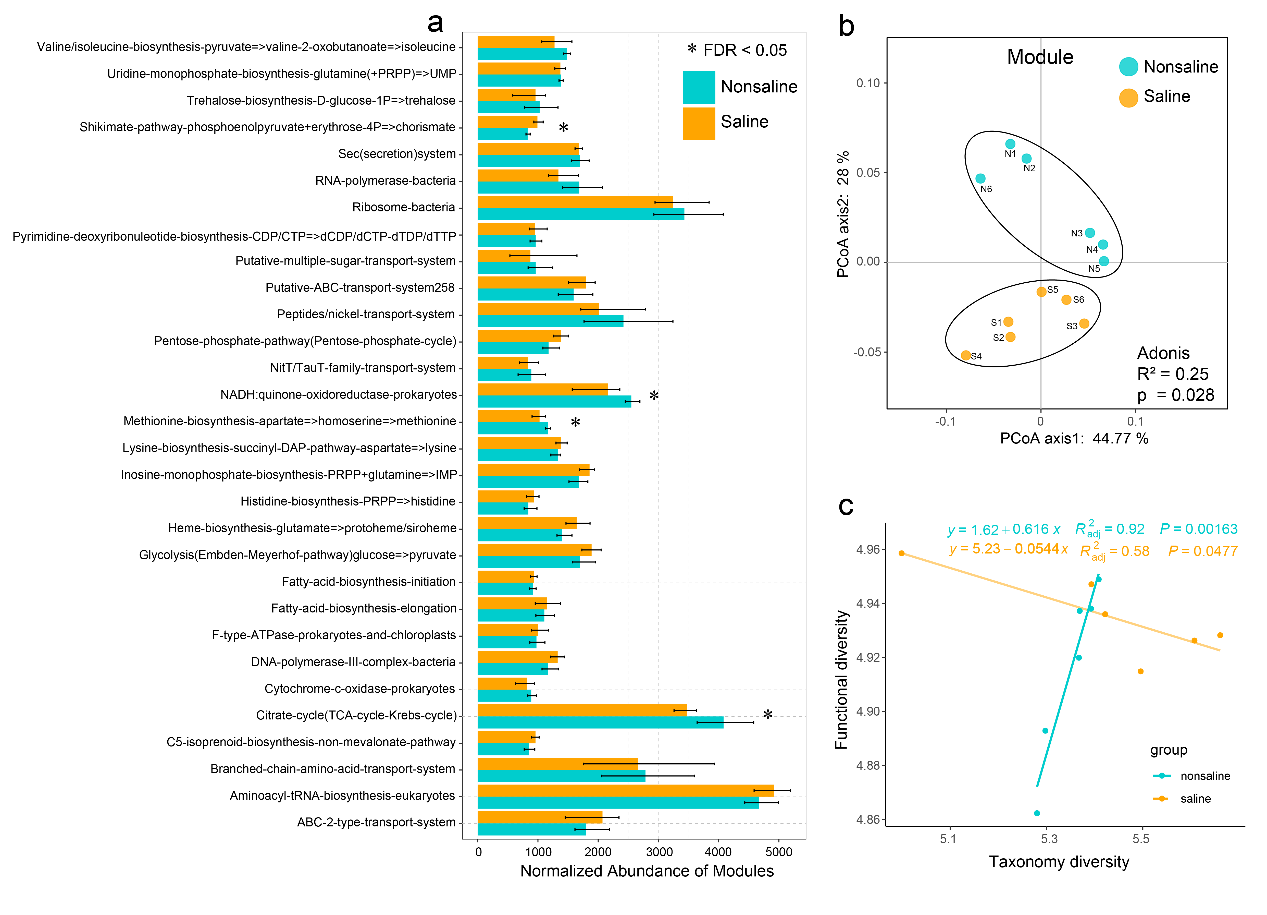


**Figure S3. The difference of bacterial community functions in saline and nonsaline soil on the Tibetan Plateau.** a. The normalized abundance of top30 functional modules in two types of soil samples from the Tibetan Plateau at module level. Significant difference (FDR < 0.05) genes are marked with “*”. The total number of reads is normalized to 100000. b. PCoA of microbial community functions at KEGG module level. The Significant differences (ADONIS, P < 0.05) of functions in the two types of soil samples from the Tibetan Plateau were tested. c. the correlation between taxonomy diversity at genus level and functional diversity at module level in saline and nonsaline soil respectively.


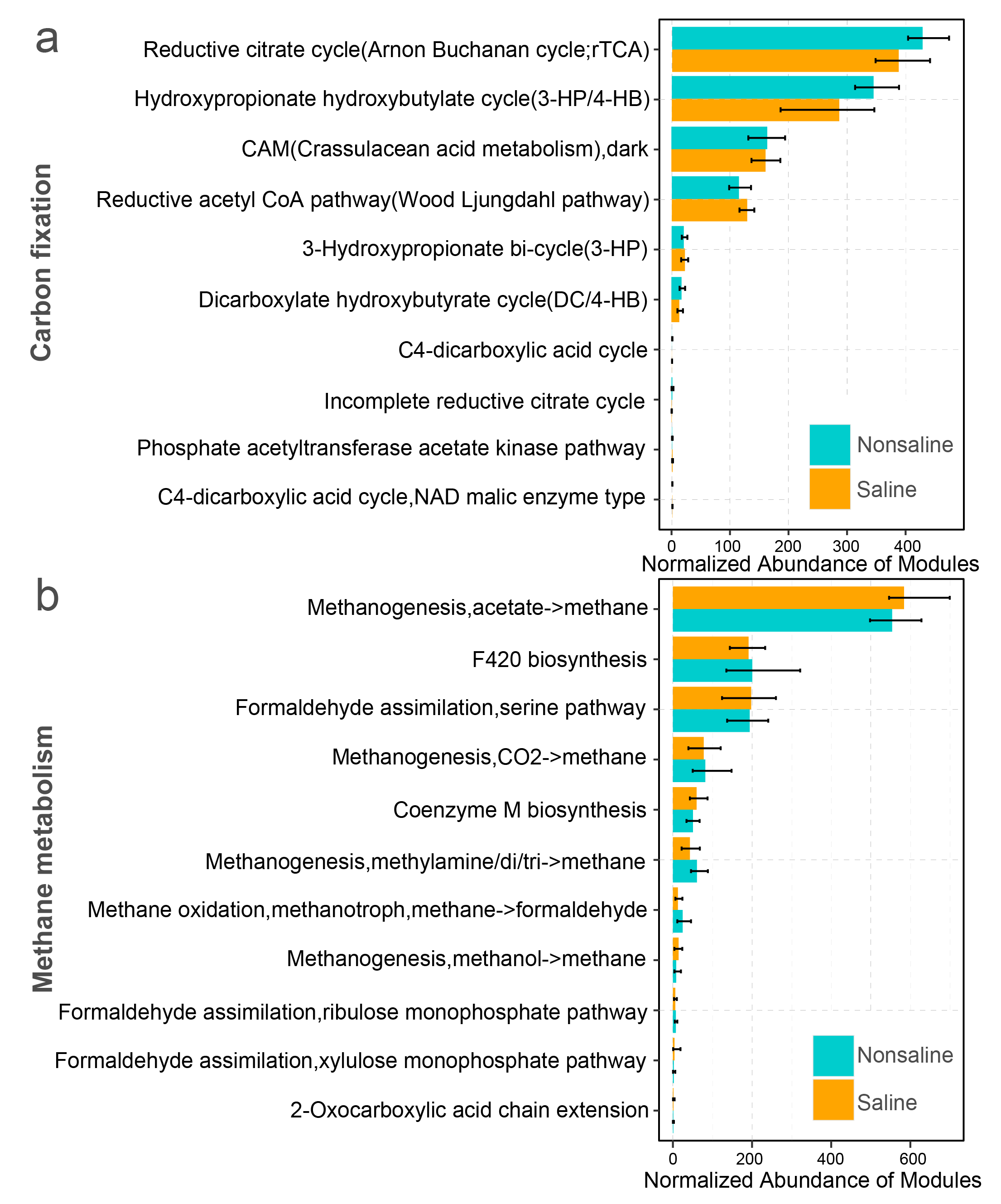


**Figure S4. Normalized abundance of functional pathways of carbon fixation (a) and methane metabolism (b) of bacterial community in saline and nonsaline soil on the Tibetan Plateau.** Bar plots show the normalized abundances of carbon cycling modules. The total number of reads is normalized to 100000.


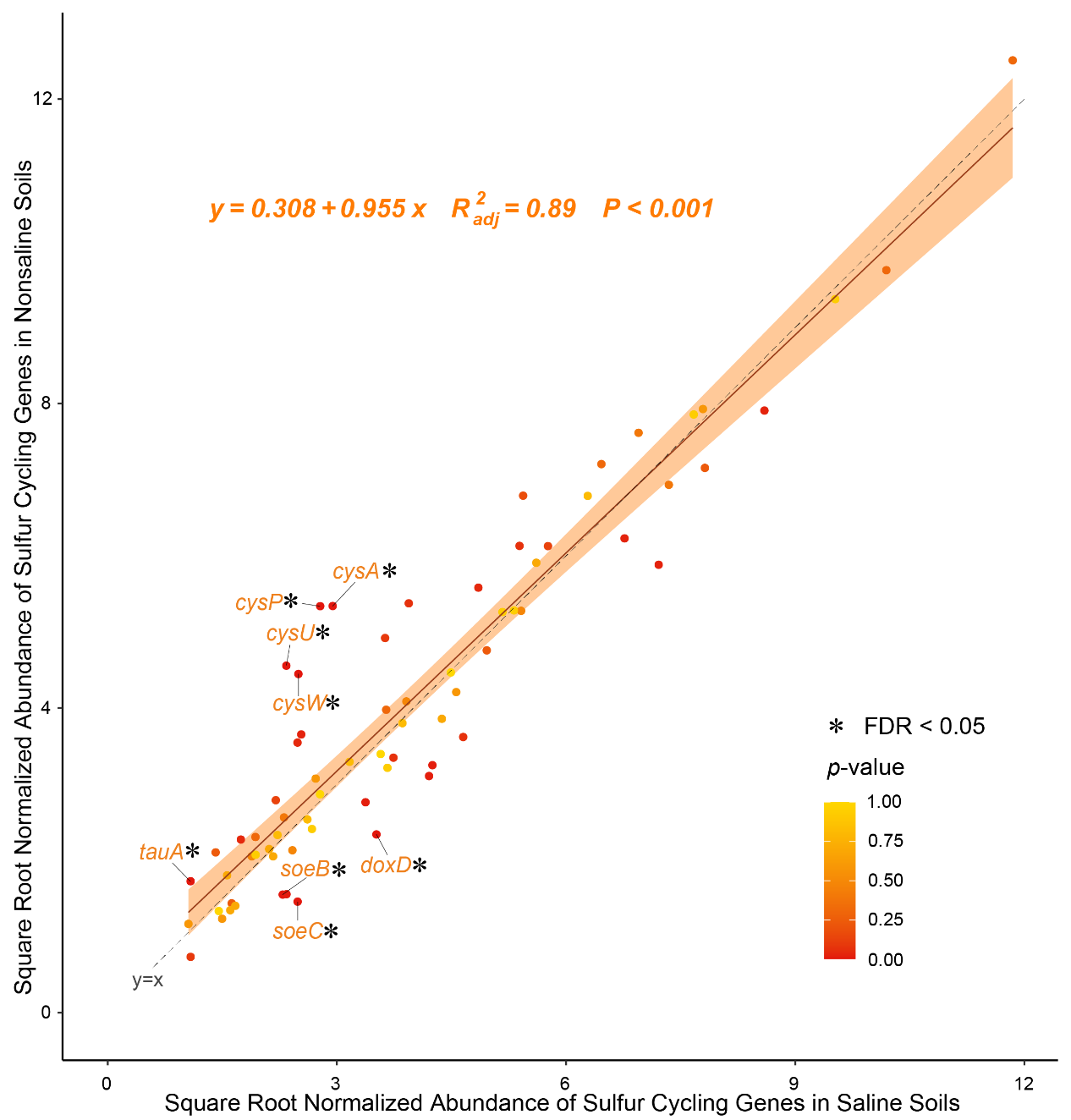


**Figure S5. The ratio of sulfur cycling genes in saline and nonsaline soil on the Tibetan Plateau.** The black dotted line equation: “y = x”, indicates that the horizontal and vertical axes are equal. Genes of Significant difference (FDR < 0.05) are marked with text in orange and connected with short lines. The total number of reads is normalized to 100000.


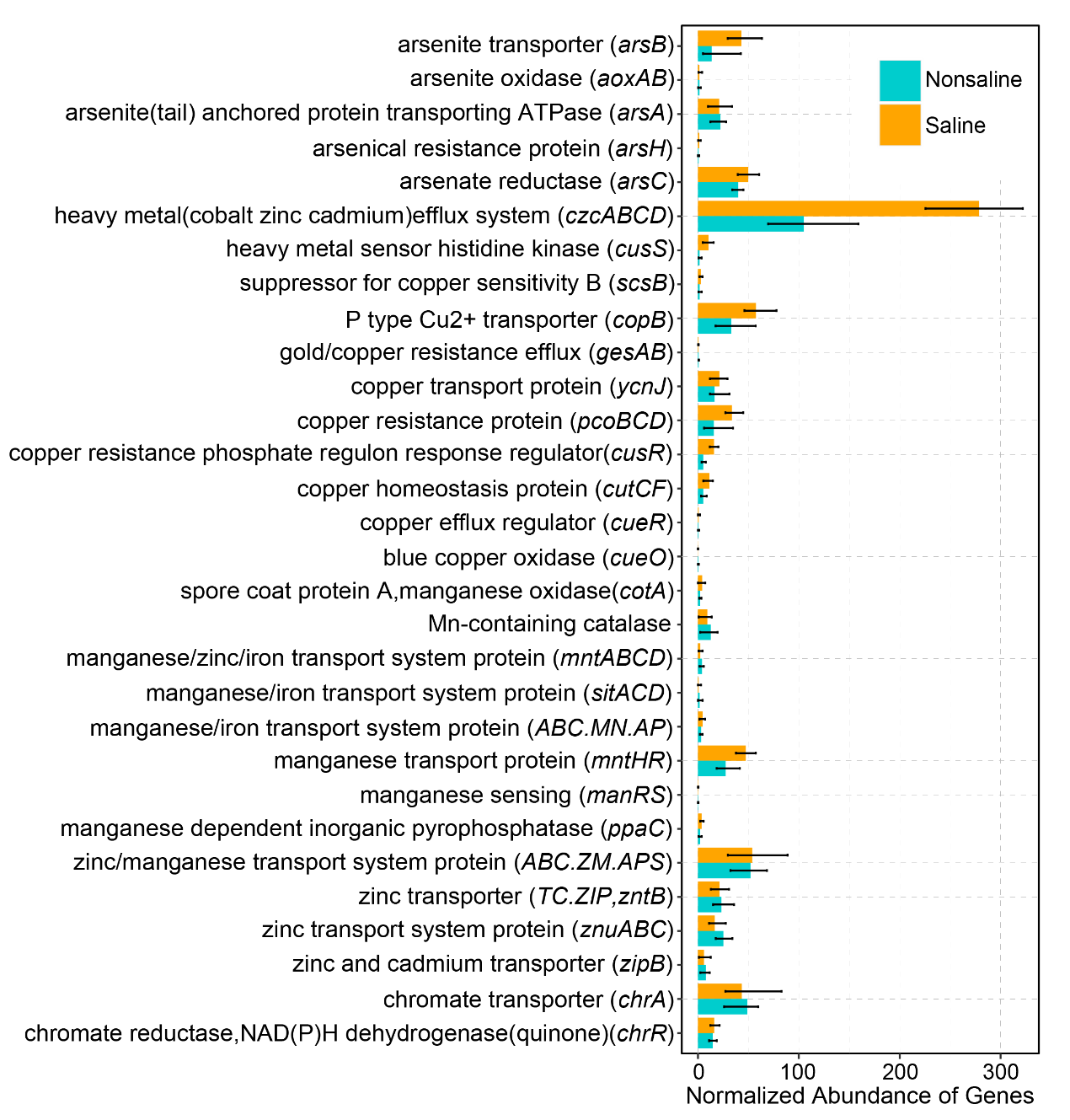


**Figure S6.** **The normalized abundance of heavy metal(loid)s resistance genes in saline and nonsaline soil on the Tibetan Plateau.** Bar plots show the normalized abundances of mental resistance genes. The total number of reads is normalized to 100000.


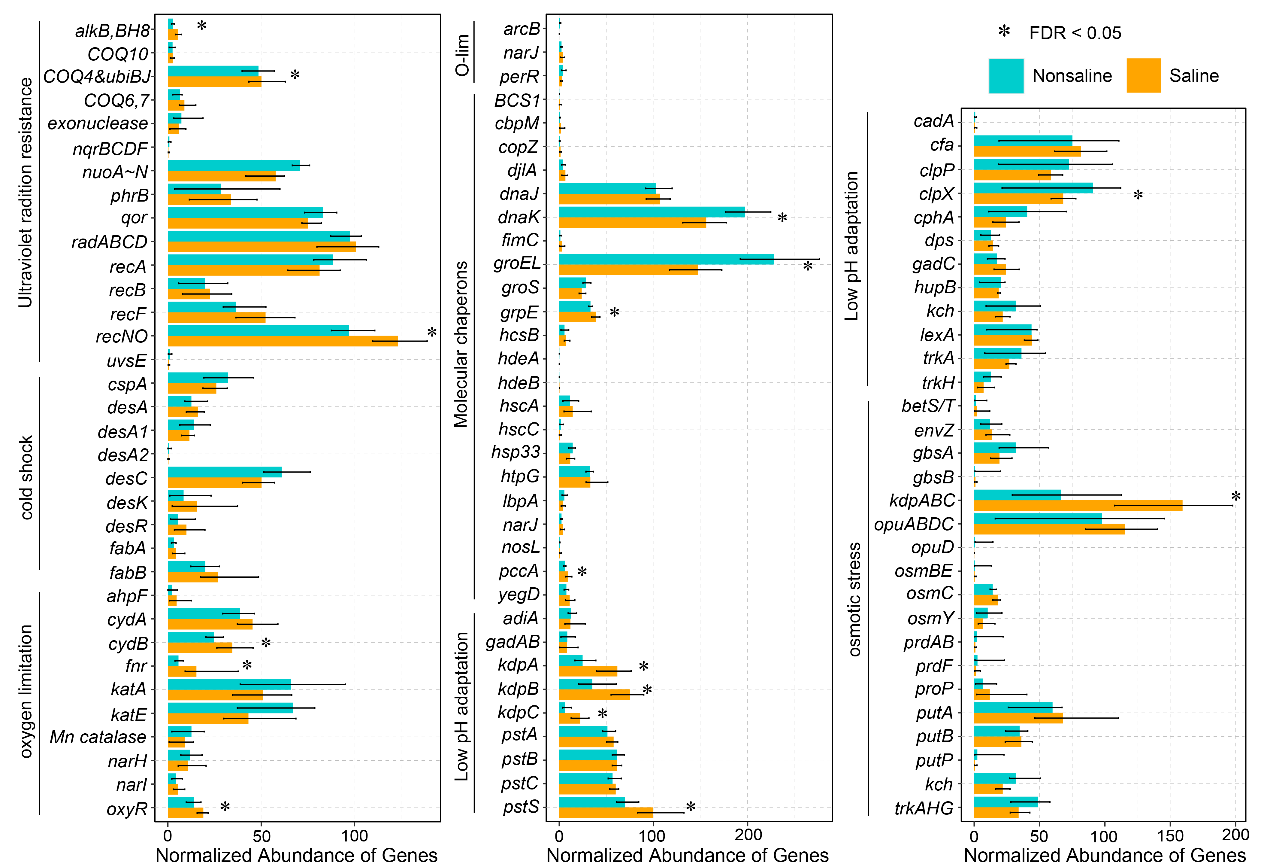


**Figure S7. The normalized abundance of environmental stress response genes in saline and nonsaline soil on the Tibetan Plateau.** Bar plots show the normalized abundances of environmental stress resistance genes. Significant difference (FDR < 0.05) genes are marked with “*”. The total number of reads is normalized to 100000.

**
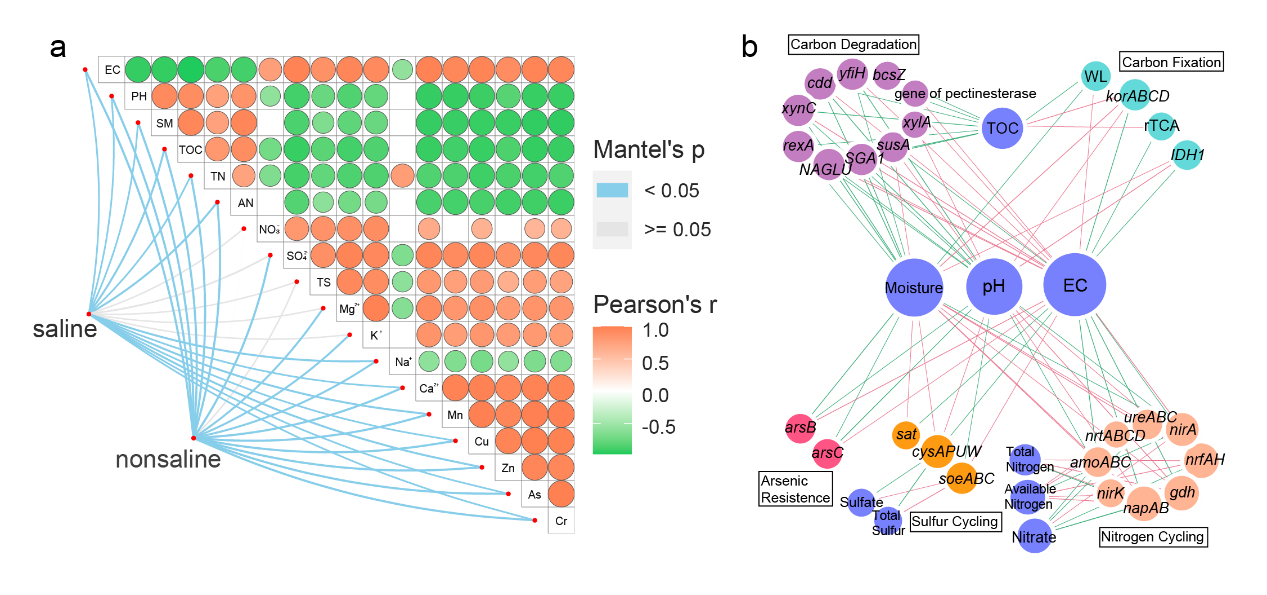
**

**Figure S8. The correlation analysis of bacterial community composition and function with environmental factors in saline and nonsaline soil on the Tibetan Plateau.**

a. The mantel correlation analysis between bacterial community composition and environmental factors. b. The significantly strong correlation (|r|>0.6, *p*-value < 0.05) between functional genes and physicochemical parameters were used to construct a network. The red and green lines mean positive and negative correlation between two nodes. The size of each node is proportion of the number of connections (i.e., degree). The thickness of each connection between two nodes (i.e., edge) is proportion of the Spearman’s correlation coeﬃcient (i.e., weight), ranging from |0.6| to |1|. WL: Wood–Ljungdahl pathway. rTCA: reductive citrate cycle.

**Table S1. The alpha diversity of bacterial community in saline and nonsaline soils of Tibetan Plateau.**

| **Samples** | **Shannon index** |
| --- | --- |
| S1 | 5.60745 |
| S2 | 5.660981 |
| S3 | 5.421845 |
| S4 | 4.999145 |
| S5 | 5.393344 |
| S6 | 5.495674 |
| N1 | 5.297761 |
| N2 | 5.40852 |
| N3 | 5.392616 |
| N4 | 5.367385 |
| N5 | 5.369099 |
| N6 | 5.280454 |

**Table S2. The topology structure characteristics of network of bacteria and environmental factors.**

| **Module** | **All node** | **All link** | **Positive link** | **Negative link** | **Color** |
| --- | --- | --- | --- | --- | --- |
| 0 | 22 | 440 | 440 | 0 | Red |
| 1  2  Environmental Factors | 42  36  9 | 598  738  219 | 594  736  108 | 4  2  111 | Lavender  Green  Blue |

Average Degree：27.266

Average Weighted Degree：20.397

Average Clustering Coefficient：0.632

Diameter：4

Radius：3

Average Path Length：1.961

Density：0.252

**Table S3. Genera in different modules in network of genus and environmental factors.**

| **Module** | | **Genus** | |
| --- | --- | --- | --- |
| 0 | *Gemmatirosa*  *Bradyrhizobium*  *unclassified_f__Acidobacteriaceae*  *Rhodanobacter*  *Candidatus_Koribacter*  *Pseudolabrys*  *Burkholderia*  *Ktedonobacter*  *Rudaea*  *unclassified_o__Rhodospirillales*  *Acidobacterium* | | *Opitutus*  *Thermogemmatispora*  *Edaphobacter*  *Candidatus_Nitrosotalea*  *Chthoniobacter*  *Mizugakiibacter*  *Dyella*  *Silvibacterium*  *Sphaerobacter*  *Granulicella*  *Dokdonella* |
| 1 | *Gemmatimonas*  *unclassified_c__Betaproteobacteria*  *unclassified_p__Acidobacteria*  *unclassified_d__Bacteria*  *Pyrinomonas*  *Candidatus_Solibacter*  *unclassified_p__Chloroflexi*  *unclassified_p__Candidatus_Rokubacteria*  *Nitrospira*  *Pedosphaera*  *unclassified_c__Gammaproteobacteria*  *Rhodoplanes*  *Haliangium*  *Pseudomonas*  *Sorangium*  *Candidatus_Entotheonella*  *Mesorhizobium*  *Steroidobacter*  *Variovorax*  *Rhizobacter*  *Polaromonas* | | *Myxococcus*  *Nitrososphaera*  *Anaeromyxobacter*  *Reyranella*  *unclassified_c__Deltaproteobacteria*  *Bryobacter*  *Microvirga*  *Massilia*  *Dongia*  *Geobacter*  *Methylobacterium*  *Labilithrix*  *Methylibium*  *Cystobacter*  *Chloracidobacterium*  *Paenibacillus*  *Cupriavidus*  *Rhizobium*  *Azospirillum*  *Herbaspirillum*  *Janthinobacterium* |
| 2 | *Sphingomonas*  *Streptomyces*  *Nocardioides*  *Arthrobacter*  *Mycobacterium*  *unclassified_f__Geodermatophilaceae*  *Leifsonia*  *Solirubrobacter*  *unclassified_c__Actinobacteria*  *Frankia*  *Conexibacter*  *Blastococcus*  *Pseudonocardia*  *Amycolatopsis*  *unclassified_o__Solirubrobacterales*  *Actinoplanes*  *Gaiella*  *Marmoricola* | | *Phenylobacterium*  *Lysobacter*  *Modestobacter*  *Phycicoccus*  *Devosia*  *Singulisphaera*  *Rhodococcus*  *Microbacterium*  *Gemmata*  *Mucilaginibacter*  *Altererythrobacter*  *Nocardia*  *Geodermatophilus*  *Rubrobacter*  *Caulobacter*  *Flavobacterium*  *Kribbella*  *Aeromicrobium* |
